# Biopolymer Composites with Sensors for Environmental and Medical Applications

**DOI:** 10.3390/ma15217493

**Published:** 2022-10-25

**Authors:** Longina Madej-Kiełbik, Karolina Gzyra-Jagieła, Jagoda Jóźwik-Pruska, Radosław Dziuba, Anna Bednarowicz

**Affiliations:** 1 Lukasiewicz Research Network—Lodz Institute of Technology, 19/27 M. Sklodowskiej-Curie Str., 90-570 Lodz, Poland; 2 Faculty of Material Technologies and Textile Design, Lodz University of Technology, 116 Żeromskiego Street, 90-924 Lodz, Poland; 3 Department of World Economy and European Integration, University of Lodz, 41/43 Rewolucji 1905 Str., 90-214 Lodz, Poland

**Keywords:** biopolymers, polymer biocomposite, sensors, environmental and medical applications of sensors

## Abstract

One of the biggest economic and environmental sustainability problems is the over-reliance on petroleum chemicals in polymer production. This paper presents an overview of the current state of knowledge on biopolymers combined with biosensors in terms of properties, compounding methods and applications, with a focus on medical and environmental aspects. Therefore, this article is devoted to environmentally friendly polymer materials. The paper presents an overview of the current state of knowledge on biopolymers combined with biosensors in terms of properties, compounding methods and applications, with a special focus on medical and environmental aspects. The paper presents the current state of knowledge, as well as prospects. The article shows that biopolymers made from renewable raw materials are of great interest in various fields of science and industry. These materials not only replace existing polymers in many applications, but also provide new combinations of properties for new applications. Composite materials based on biopolymers are considered superior to traditional non-biodegradable materials due to their ability to degrade when exposed to environmental factors. The paper highlights the combination of polymers with nanomaterials which allows the preparation of chemical sensors, thus enabling their use in environmental or medical applications due to their biocompatibility and sensitivity. This review focuses on analyzing the state of research in the field of biopolymer-sensor composites.

## 1. Introduction

Biosensors are diagnostic tools used in many areas of life: medicine, the environment, the packaging industry and many others [1,2,3,4,5]. The combination of sensors with biopolymers and natural polymers, which are a natural and sustainable alternative to petroleum-based polymers, is particularly noteworthy. Biosensors and chemical sensors are monitoring devices that interact with various chemical or biological components to provide valuable information [1].

Modern sensors are characterized by high sensitivity, selectivity, on-time response, stability, reversibility, are low-cost, and are easy to use. Their great advantage is the quick reaction to the presence of the monitored factor, which allows for quick intervention.

The development of chemistry and materials engineering creates enormous opportunities to develop new solutions, the application of which in everyday life is a milestone in health protection, the environment, food waste, agriculture and many other areas [1].

Chitosan, alginate, cellulose and starch are some of the natural polymers that are widely used in conjunction with sensors. The wide range of applications of biopolymers results from their properties, such as their capability to collect and accumulate analytes on sensor surfaces, and the possibility of producing and using various forms (film, sponge, hydrogel). 

**Figure 1:**
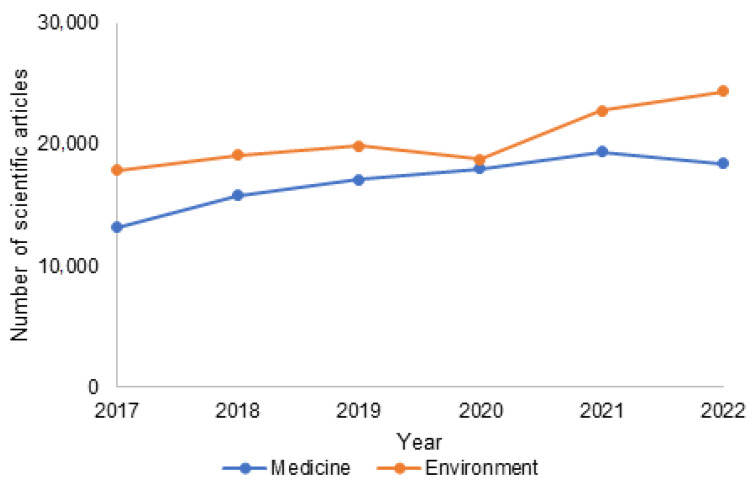
Number of scientific articles focus on the use of polymers as sensors in environmental and medical research. Keywords: biopolymer and sensor and (blue) medicine; (orange) environment (data from google scholar; accessed October 2022).

The scientific interest in the use of biosensors in combination with biopolymers in many fields of science is growing every year. Figure 1 shows the number of scientific reports related to the use of sensors in environmental research and medicine. The growing number of scientific reports proves not only the great potential of this direction but above all the real need of developing modern solutions for monitoring selected parameters. Both the basic and implementation works constitute a huge substantive contribution in this field and have become a signpost for the search for new concepts.

This paper presents an overview of the current knowledge on biopolymers in combination with biosensors in terms of properties, methods of linking and applications, with particular attention to the medical and environmental aspects. The current state of knowledge as well as future perspectives were presented. 

## 2. Biopolymers and Their Composites

Polymers play an important role both in the biological world and in its present industry. Many natural polymers, for example proteins and nucleic acids, transfer fundamental biological data, whereas other polymers such as polysaccharides contribute fuel for cell functions and serve as constructive elements in living structures [1]. Biopolymers can be used in various applications such as food packaging, cosmetics and medicine, biomimicking, actuators, tissue engineering, sensors, and ultrasound imaging [6]. Recently, there has been a significant intensification of research works on biodegradable polymers and their composites. This phenomenon is associated with the gradual depletion of crude oil deposits and problems related to the management of used non-biodegradable plastics.

Biopolymers are materials that have potential applications in all sectors of the economy. Polymer composites are biocomposites if at least one constituent is biobased or biodegradable. The terms biodegradable polymer and biopolymer are often used interchangeably in the literature, but there is a major difference between the two types of polymers [7] that undergo deterioration and completely degrade when exposed to microorganisms, aerobic, and anaerobic processes [8]. Biobased is a term applied to polymers originating from renewable sources. Raw materials are defined as renewable if they are replenished by natural procedures at rates comparable to or more rapid than their rate of utilization. The classification of “biopolymers” can be made considering two criteria:

- The source of the raw materials; 

- The biodegradability of the polymer.

**Figure 2:**
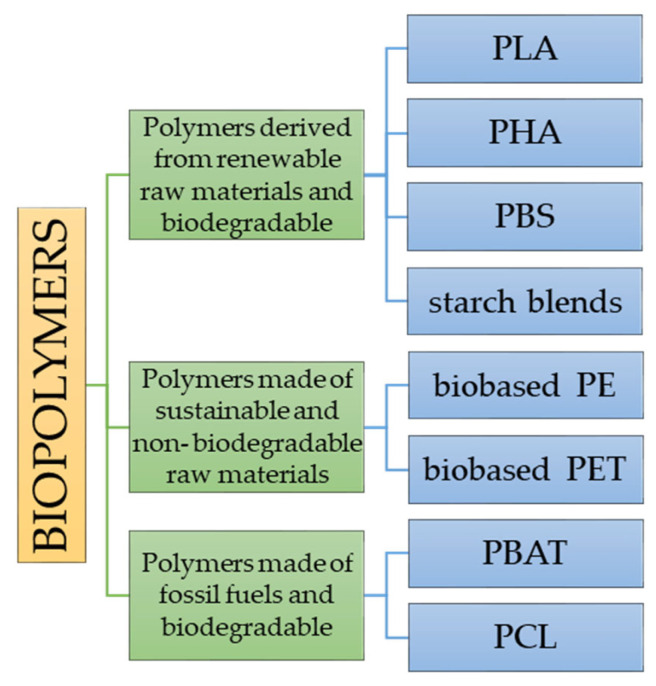
The division of biopolymers based on the origin of the raw material and biodegradability [9].

Considering the two criteria for the division of polymer compounds, i.e., the source of origin of raw materials and their biodegradability, the compounds shown in Figure 2 can be described as a biopolymer. 

**Figure 3:**
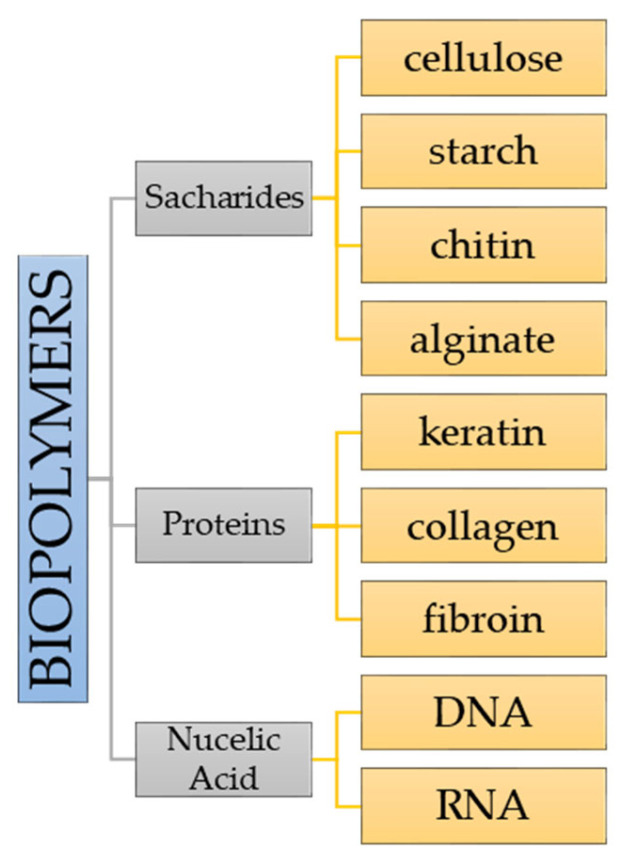
Division of biopolymers based on monomeric units.

A different approach regarding the division of biopolymers is according to monomeric units. It is possible to differentiate three different groups (Figure 3) [10,11].

When the monomeric unit consists of polysaccharides, there are carbohydrate chains, which can be branched or linear joined by glycosidic linkage. Examples of such monomeric units are starch and cellulose. Protein units are made up of amino acids which can be represented by collagen or fibrin which are linked with peptide bonds (amide linkage). The third group, which is a protein monomeric unit, consists of nucleic acids which can be differently arranged in long polymer chains made of at least 13 units. Examples are DNA and RNA [12]. 

Biopolymers can be produced both by biological organisms, e.g., plants and animals, and chemically synthesized from natural starting materials, e.g., corn, starch, etc. A detailed classification of the biopolymers was discussed by Dziuba et al. [13]. Biopolymers are important materials for the production of composites with sensors used in many areas of the economy and industry. The perfect performances of biopolymers from biological chemical sensing systems are perfect candidates for sensitive elements for developing sensors using biopolymers for chemical sensing in complex environments. Sensors using biopolymers/biomaterials for chemical sensing have reached meaningful progress and shown helpful perspectives and potential applications. When designing composite materials, an extremely important issue is the selection of an appropriate polymer matrix. Increasingly, polymers used as matrices are biodegradable. One of the major advantages of biodegradable polymers contributing to their growing popularity is the relatively easy biodegradation of such materials. Thanks to the use of various additives (e.g., starch or montmorillonite) it is possible to control the biodegradation time of the material [14].

**Figure 4:**
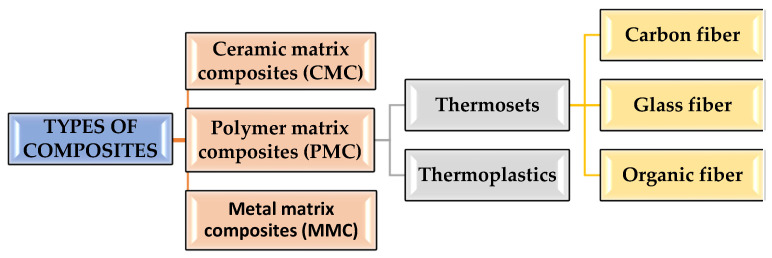
Classification of polymer matrix composites.

Composites are materials that comprehend two or more chemically and physically diverse phases isolated by a separate interface. They are a mixture of materials differing in composition, where the particular components maintain their unique identity. Summary composite materials are a combination of two or more components, one of which is present in the matrix phase and the other may be in the form of particles or fibers. Figure 4 shows the types of composite materials depending on the polymer matrix and Table 1 depicts the polymer composites with different reinforcements and matrix materials.

CMCs have high creep resistance and thermal shock resistance. CMCs are fabricated by hot pressing, hot isotactic pressing and liquid phase sintering techniques and are used as cutting tool inserts for machining hard metal alloys with a tool life greater than cemented carbides. Ceramic composites improve the fracture durability of material [15,16]. PMCs are intended to carry loads in between filaments of a matrix material [17,18]. 

Advanced composites of fibers and grid combinations yield superior quality, mechanical strength, thermal stability, hardness and stiffness [19,20]. Glass fiber-reinforced polymers comprised of fibers between 3 and 20 mm in a polymer network [17,21]. Carbon fibers have the highest quality and modulus at higher temperatures [22], while at room temperature, carbon fibers are not influenced by wetness, bases or acids [23].

**Table 1:** Detailed literature review of polymer matrix composites.

Matrix Material	Reinforcement	Findings	Literature
Thermoplastic composites	Carbon fiber	Increased tensile strength and stiffness, improved printing versatility	[24,25]
Polylactic acid	Carbon fiber	Increased Tensile strength, Young’s modulus and yield strength at layer thickness was 0.15 mm	[26]
Virgin polypropylene and Polypropylene with 30% Glass fiber	Glass Fiber	Virgin polypropylene has better stability at multiaxial stresses than PP30GF	[27,28]
Polylactic acid	Water in oil emulsions and hydrophobic polymers	PLGA made the sheets more flexible because PLA is crystalline, whereas PLGA is amorphous, improved biocompatibility	[29,30,31]
Polylactic acid	Almond skin powder	Increased compressive strength and shore D hardness	[23]
Poly butylene succinate	Modified tapioca starch	Dispersion rate of starch molecules is low, Adhesive properties poor, and lower mechanical properties due to high voids in the structure	[32,33]

These differing elements act together to produce the essential mechanical strength of the composite part [34]. A special group of polymer composites is biocomposites. Biocomposites are polymer composites, in which at least one constituent is biobased or biodegradable [35]. Biocomposites include natural reinforcements (such as vegetable fibers) in their mixture and can be: (1) partially biodegradable with nonbiodegradable polymer matrices such as thermoplastic polymers (e.g., polyethylene) or (2) fully biodegradable with biodegradable polymer matrices such as renewable biopolymer matrices. 

The advantage of composite materials is the ability to design a range of properties that may vary depending on the intended use of the composite. The key advantage of these materials is their programmability, which now allows them to be widely used throughout the world around us and in various areas of life, such as construction, industry, medicine, production of everyday objects, and even devices used in space. 

Today, biocomposites and biopolymers with controllable lifetimes have become major topics for different areas of applications. This article focuses on the use of biocomposites along with sensors for environmental and medical applications.

## 3. Biosensors

A key question to be answered is what a biosensor is. This is a tool that can be used for obtaining the physical identity, which is then translated into a recognizable signal. It should have the ability to react with different biological components to react in the desired way. The biosensor can detect for example biomolecules such as urea, estrogen, glucose, or cholesterol [36]. 

Eight major parameters characterize the biosensor:

1. Sensitivity that is for example the concentration of the influencing element;
2. Selectivity, so the sensor will not react to every molecule but just the expected one;
3. The range is strictly connected with sensitivity and corresponds to the concentration range where the sensitivity is active;
4. Response time, how much time is required for a biosensor to indicate 63% of the final response;
5. Reproducibility, the accuracy of the sensor’s output,
6. Detection limits its lowest detectable concentration;
7. Lifetime is the period when the biosensor can be used without significant loss in performance;
8. Stability, if within a fixed period there is any change in the baseline or sensitivity [37].

**Figure 5:**
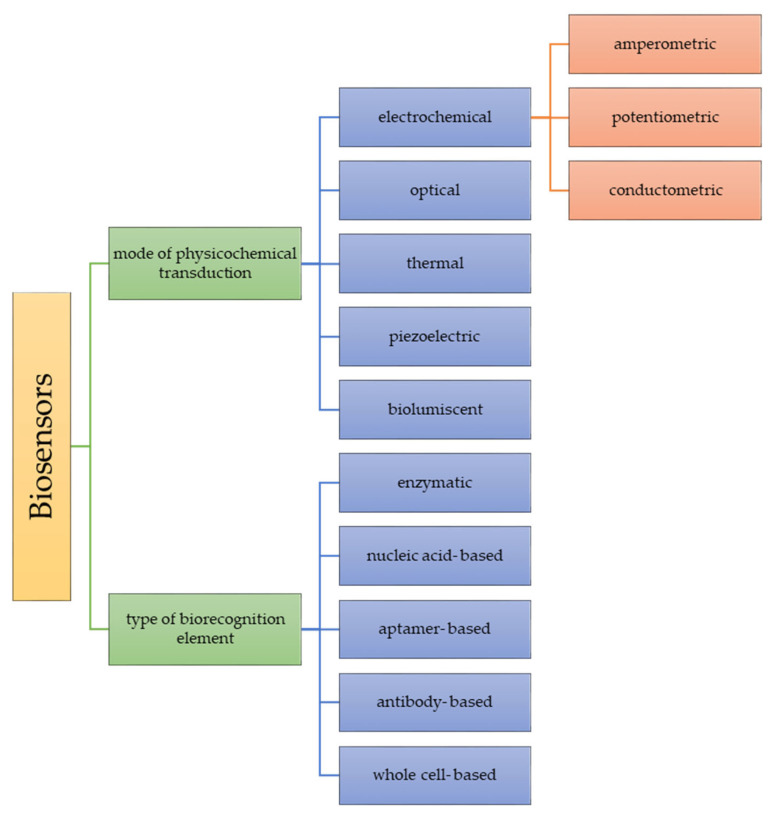
Classification of biosensors [38].

The classification of biosensors can be based on the mode of physiochemical transduction or the type of biorecognition element (Figure 5) [38,39,40].

Electrochemical biosensors are the most widely described and investigated due to their properties. They can be characterized by a low detection limit, simplicity of construction, specificity and ease of operation [41]. 

The operation of optical biosensors is based on the occurrence of a biochemical reaction, which is connected with light absorption or emission. Absorption, luminescence, fluorescence, surface plasma resonance, etc., are among the techniques linked to this group of sensors. The visible color change makes colorimetric sensors the most explored [42]. 

Because most of the biochemical reactions involve a change in enthalpy and heat changes, it is reasonable to use the phenomenon in biosensor design [38]. 

Piezoelectric biosensors use piezoelectric crystals that participate in the measurement of the mass change by correlating with the change in oscillation frequency. This phenomenon is a result of biomolecular interaction [38]. 

A special class of biosensors is conducting polymers (CPs), which contain large resonation structures with many sp2- carbon atoms that enable the delocalized transport of charge carriers. Synthetic metals such as polyaniline (PANI), polypyrrole (PPy), polythiophene and derivatives are among the group [43,44]. 

Biosensors can also be classified based on the utilized transduces. Here, three generations can be distinguished. The first one includes biosensors, which use electrical responses that occur through the diffusion of reaction products to the transducers. A high-applied potential and fluctuant concentrations of the product, which can result in interferences, decrease in electrical currents and limits of detection, are among the main limitations of this group. The separation of the second generation was directly related to the application of mediators between the reaction products and transducers. The lack of selectivity of the mediator results in various interfering interactions. The third generation of biosensors revolves around the biochemistry and electrochemistry that occur as a semiconductor. This category is characterized by high selectivity and sensitivity [45,46].

Biosensors can be used in various applications shown in Table 2.

**Table 2:** Applications of biosensors.

Parameter to Monitoring	Literature
Pressure sensing	[47,48]
Temperature sensing	[49,50]
Monitoring of neurological function	[51,52]
Rehabilitation and physical therapy	[53,54,55]
Cardiopulmonary and vascular monitoring	[56,57]
Glucose monitoring	[58,59]
Humidity sensing	[60,61]
Detection of homocysteine biomarkers, carbohydrates, bioactive thiols, phenols, catechols, lactate and ethanol, dopamine, nucleotides, DNA, cell behavior and morphology	[62,63,64,65,66,67,68,69,70,71,72,73,74,75]
Heart diagnosis (cardiovascular diseases)	[76,77]
Detection of the outbreak of virus	[78,79]
Retinal prostheses	[80]
Enzyme biosensors	[81,82]
Phenotypic diagnostic for cancer	[83,84]
Rapid DNA and RNA diagnostic	[85,86]
Medical mycology	[87]
Optical DNA diagnostics	[88,89,90]
Overall health monitoring	[50,91]
MRI contrast imaging	[92,93,94,95]
Food safety	[81,96,97,98]
Sustainability	[99,100]
Quality of products	[65,101]
Abiotic stress	[102,103]
Plant infections	[104,105]
Phytohormones	[106,107]
Metabolic content	[65,108]

Bioresponsiveness is a property that allows the polymer to respond selectively to given molecules. 

An example of such behavior is chondroitin sulphate, which is a glycosaminoglycan and can bind to CD44 receptors which are commonly expressed in cancer cells. This can be used in recognition of the kind of cells as well as the attachment. What is more interesting is that the biopolymer undergoes degradation when in the environment where there is the presence of the enzyme lysosomal hyaluronidase, which is also commonly present in cancer cells. The ability to attach and degrade may be used in the targeted treatment of cancer cells by delivery of a drug. 

Similar behavior can be used for the diagnosis of wound infections. For this purpose, alginate or agarose matrix was used and peptidoglycan was covalently bound with Remazol, which is a blue dye. Primarily the matrix is yellow, but in the presence of lysosome and elastase in wound fluid hydrolysis occurs, releasing dye which changes color from yellow to blue. 

Next, interesting research was conducted by Wang et al. [109] and it was dedicated to sensing ATP molecules by ATP aptamer-capped gold nanocage filled with Rhodamine B dye molecules. ATP molecules can be detected because they trigger displacement stimuli.

T of ATP aptamer caps from the surface of gold nanocage, in this way causing the opening of pores and releasing enclosed dye inside, causing a visible change of color. The important thing is that the treatment has high selectivity as it does not respond to ATP derivatives such as GTP, UTP, or CTP. The ability can be used for detecting cellular ATP in Ramos cells or in situ living Ramos cells. 

Schneider et al. [24] conducted experiments devoted to different types of bioresponsive material, which is considered a detection tool for the presence of bacteria or fungi. For this purpose, the Alizarin dye was trapped in the hydrogel made of carboxymethyl cellulose and polygalacturonate. Detection worked in the following way, the pathogenic microorganism caused hydrolysis of carboxymethyl cellulose and in the same way, the degradation of hydrogel, triggering the release of dye. 

A biosensor devoted to the sensing of glucose was also tested. It is based on immobilizing glucose oxidase into a polymeric carrier. This enzyme converts glucose into gluconic acid, which decreases pH and consequently protonation of the polymer chain. Thanks to coulombic forces, swelling occurs, releasing entrapped dye. This behavior may be used in the detection and drug delivery. The mentioned treatment may be considered for diabetes for the detection of glucose in the blood. 

Carbon-based biopolymer composites offer interesting properties in the biosensing field. Their properties include high surface area, conductivity, efficient electron transfer, and so on. An example of using the carbon in biosensors is biopolymer–MWCNT (multiwalled carbon nanotubes) when deposited enzyme organophosphorus hydrolase–MWCNT and anionic DNA–MWCNT allows sensitive detection of paraoxon. Graphene with enzymes when dispersed in biopolymers (for example chitosan) and used with some negatively charged polymer may form a film on the electrode and subsequently allow the reagentless detection of H_2_O_2_ [38].

## 4. Linking of Biosensors and Biopolymers

The proper functioning of a biosensor is directly related to the preservation of the activity of its biological moiety. The selection of a matrix that ensures the immobilization of sensors and biocompatibility is of great importance. Biopolymers have properties that make them a great candidate for this purpose. A wide range of biopolymers can swell in an aqueous environment, which helps to eliminate the diffusion barrier for the analyte. Additionally, the preparation of biopolymer-based composites is relatively easy and improves their properties [38]. 

**Figure 6:**
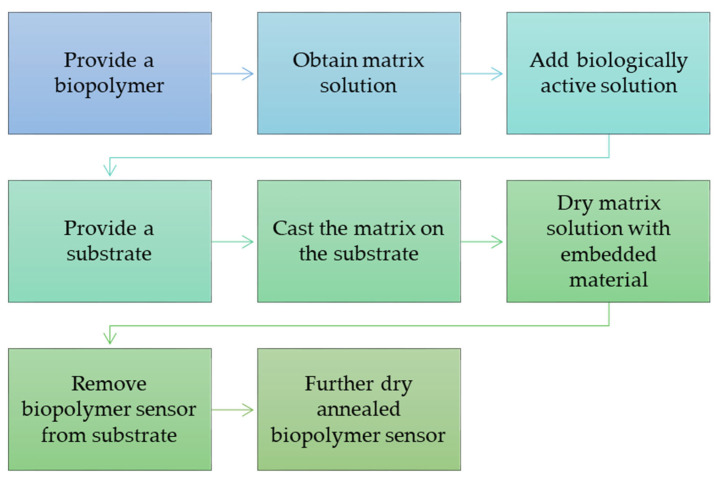
Scheme of the connection of biopolymers with sensors [110].

The process of bonding the biopolymer to the sensors follows the sketch below (Figure 6).

The biological recognition element is the key unit of biosensors. It is responsible for the interaction with the target analyte to produce the desired signal. There are many methods to connect the sensor to the matrix. One of them is immobilization [38,111], which uses physical adsorption, covalent binding, physical entrapment, and cross-linking. A wide range of analytical techniques has been used to determine the presence of various pollutants. However, usually, their use is associated with the necessity of transporting the sample to the laboratory for analysis. Therefore, there is a need to develop fast, portable environmental monitoring systems. Biosensors seem to be a great solution, which can be seen as an alternative or complementary method for analyte detection. A biosensor is defined by the International Union of Pure and Applied Chemistry (IUPAC) as a self-contained integrated device that is capable of providing specific quantitative or semi-quantitative analytical information using a biological recognition element (biochemical receptor), which is retained in direct spatial contact with a transduction element [112].

## 5. Biosensors for Environmental and Medical Applications

### 5.1. Environmental Application

Caring for the natural environment and its safety should be one of the main priorities of today’s society. Its condition directly affects human health. Yet, the number of potentially harmful pollutants indicates that this is still a serious problem that must be dealt with. Due to this fact, both the detection and monitoring of pollutants in each component of the environment are crucial for the safety of humans, animals and plants [112]. 

The main application of biosensors in agriculture and the environment is connected with monitoring and determination of the presence of contaminants and infectious illnesses. Biosensors can be used in the detection of the following: water and air pollutants; pesticides for agricultural products; and radiation. In the food sector, biosensors can be applied in the monitoring of food safety, sustainability, and quality of products [113]. 

Literature reports that the development of nano-inspired biosensors makes a huge contribution to the understanding of molecules whose dynamics have a remarkable influence on plant physiology [114,115]. The development of various biosensors for detecting abiotic stress, plant infections, phytohormones, metabolic content, and miRNA has been described [103]. 

#### 5.1.1. Environmental Pollution

Biosensors have found applications in the detection of environmental pollution. The main sources of pollutants (both organic and inorganic) present in the environment are industry, agriculture and others related to human activity [116]. Biosensors are used to monitor the presence of pesticides, potentially toxic elements, toxins, pathogens, and endocrine-disrupting chemical compounds in water, air, and soil [117]. Scientists’ attention is focused on the quantification of long-lasting toxicants. The main features of biosensors used as biodetection devices are the possibility of their reuse and resistance to pH and temperature.

Biological materials, including enzymes, microbes, functional nucleic acids, antigens, antibodies, animal and plant tissue, and biomimetic materials (e.g., molecularly imprinted polymers) are among the elements responsible for biorecognition [118]. 

Intensive use of some chemicals prompts reflection on the environmental effects. Among these substances are tetrabromobisphenol A (TBBPA) and its derivatives, which are widely used as brominated flame retardants (BFRs). The derivative tetrabromobisphenol A bis (2-hydroxyethyl) ether (TBBPA-DHEE) aroused great concern due to its potential neurotoxins and high toxicity [119]. Its determination is possible with analytical instrumental methods and immunoassay. Zhang et al. [120] reported the application of ultrasensitive competitive impedimetric immunosensors to simultaneously detect TBBPA-DHEE and TBBPA-MHEE. The proposed system was based on the analyte, coating antigen (coated on glassy carbon electrode (GCE)/chitosan (CS)/MWCNTsGONRs/gold nanoparticles (AuNPs), modified electrodes) and primary antibody (Ab1). The biosensor can be used in the monitoring of the aquatic system. Monitoring water pollution is of great importance, due to the water cycle and its relevance in our daily lives. The scientists [121] described an easy-to-use biosensor composed of disposable bioreporter pads (calcium alginate matrix with immobilized bacteria and non-disposable photodetector. The authors examined the effect of alginate concentration, viscosity and bacteria density on the sensor response. Its response was checked in the presence of several common and environmental chemicals at different spiked concentrations (e.g., heavy metals, ammonium hydroxide, formaldehyde). The optimized product was characterized by low detection limits, small amounts of a sample, and high sensitivity.

#### 5.1.2. Pesticides 

Pesticides, including herbicides, are a huge threat to environmental and whole ecosystem safety. The list of herbicides is long, and special attention should be put to photosynthesis inhibitors, such as urea, triazines, and phenolics. They often influence non-target habitats, such as neighboring vegetation and freshwater ecosystems [122], [123]. Literature [124] reports that herbicides can act as endocrine disruptors, especially in cases of long-term exposure. Biosensors are a great alternative to traditional analytical methods such as HPLC, AAS and GC-MS, which are time-consuming and expensive. The researchers [125] presented a novel amperometric biosensor based on direct inhibition of the photocurrent generated by an artificial biofilm of photosynthetic microorganisms. The sensor was designed with the use of living cells, which were encapsulated in an alginate matrix, attached to the carbon-felt electrode. The immobilization of bacterial cells (*Anabaena variabilis*) in alginate capsules resulted in a high cell density crucial for bacterial stability. 

Trichloroethylene (TCE) is one of the common pollutants of groundwater and soil that is uneasy to degrade by microorganisms. The literature [126] reports on the development of a microbial biosensor based on the Pseudomonas sp. strain, which can detect the compound. A porous cellulose nitrate membrane was used to immobilize the bacteria. A chloride ion electrode was employed as the transducer [127]. The research involved the examination of different concentrations of TCE and bacteria, pH levels, temperatures, and interferents. The biosensor was composed of *Thiobacillus thioparus*, used as the recognition element, which was immobilized on sodium alginate and an agarose bed. Oxygen reduction was considered the detection sign. The authors confirmed that the obtained final biosensor had a satisfactory value of oxygen taken up by the immobilized cells.

#### 5.1.3. Wastewater

Monitoring the wastewater is also of great importance. The whole-cell electrochemical biosensor was described in the detection of heavy metal ions (Cu^2+^, Cd^2+^, Ni^2+^, Pb^2+^) [128]. The sensor consisted of a chitosan hydrogel polymer film with boron-doped nanocrystalline diamond particles, which was electrodeposited onto a glassy carbon electrode to immobilize Saccharomyces cerevisiae cells and the mediators. The research revealed the possibility of using the developed indicator in the assessment of acute toxicity of real wastewater samples, which is promising in the online detection system. Wastewater monitoring can also be conducted by the use of electrochemical biosensors with dye mediators. Fang et al. [129] proposed a solution based on the application of an organic dye mediator (thionine) that uses electrostatic interactions to wrap *E. coli*. These bacteria are the most common Gram-negative bacterium widely used for toxicity assessment of chemicals. *E. coli* was immobilized into chitosan-entrapped carbon nanodot film and further modified on a glass carbon electrode. The use of this electrode increased the conductivity and improvement of electron transfer. Another biosensor used in the assessment of heavy metals (Cu^2+^, Cd^2+^), phenol (3,5-dichlorophenol), and pesticides were developed using a p-benzoquinone-mediated whole-cell electrochemical biosensor. The action was based on the coimmobilization of mixed strains of microorganisms (*E. coli, B. subtilis, S. cerevisiae*). The microbial biofilm was prepared with the use of sodium alginate solution. The conducted study revealed that the sensor can be successfully used in the assessment of ecological risk [128].

#### 5.1.4. Air Toxicity 

Another component whose quality must be controlled is air. Calcium alginate pads with immobilized bacteria (*E. coli*) were used to prepare biosensor that enables monitoring of air toxicity. The researchers [130] applied glue spray, bleach, oil strain remover, Tipex, paint, fuel, weed killer, chloroform, and acetone as toxicants. The presence of all these chemicals simulated cell response. The sensor demonstrated the ability to sense the presence of chemicals in a real, indoor environment. 

Despise biopolymers, synthetic polymers are also widely used in environmental control. Literature reports about their application in the detection of inter alia pesticides [131], and heavy metals in soil [132].

### 5.2. Medical Application

The dynamic developments in medical material engineering enabled the design of modern functionalized wound dressing. This is an important direction due to the aging population, which requires modern methods of treatment. The history of the dressing goes back about 2100BC. The famous Sumerian clay tablet contains procedures for dressing wounds using bandages [133,134]. Ancient Egypt also produced a medical document, THE EDWIN SMITH PAPYRUS, which was written around 1700 BC, but most of the information is based on texts written around 2640 BC—Imhoteps time. This document is one of the oldest known medical papyruses. It contains the first descriptions of surgical sutures and various types of dressings [135,136]. 

It was common to use medical products in ancient Rome and Greece. The first figure who brought much new knowledge to medicine, including Roman surgery, was Aulus Cornelius Celsus (30 BC—45 AD), author of a critical encyclopedic work DE MEDICINA LIBRI OCTO. The second most important figure in ancient medical thought was THE PERGAMON OF AELIUS GALEN (129–201 AD), who described the use of silk in 150 AD. 

In ancient Greece, medicine developed mainly due to the works and writings of Hippocrates. Medicine reached a particularly high level in ancient India. Abundant reports show that dressing materials (cotton, silk and linen) are common [137]. The Indian surgeon Sushruta developed suture materials based on natural fibers from linen, hemp and hair [138]. The Johnson & Johnson Company (New Brunswick, NJ, USA) began mass production of sterile surgical dressings in 1891 by sterilizing cotton and gauze by dry sterilization and then by steam and pressure [134]. 

Currently, medicine has reached a high level in terms of wound dressings, e.g., hydrogel dressings made from hydrophilic, inflatable gel or film, polysaccharide foams, flexible fibers, and herbal dressings [139,140,141,142,143]. The topic is still very developmental. New technologies are of particular importance, especially for difficult-to-heal and burn wounds. Development in this technological area is a highly important activity because of the aging society, which requires high-level medical care. This problem concerns not only Europe but the entire world population. The forecast of the United Nations predicts that by 2030 the percentage of Europe’s population over 65 will be 23.8%. Moreover, EUROSTAT data show that the relative size of the elderly population is growing faster than any other age segment of the EU population. The proportion of people over 80 years old in the population of the EU- increase between 2019 and 2100 from 5.8% to 14.6%. The problem of aging populations in the coming decades will also apply to underdeveloped countries. 

**Figure 7:**
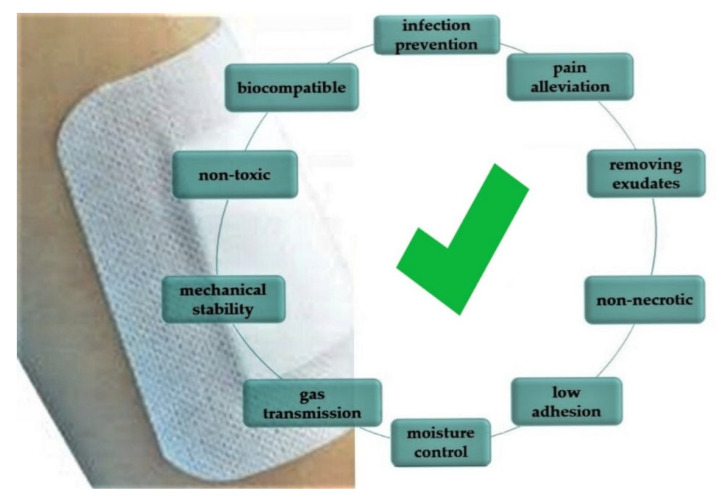
The most important requirements for wound dressings.

According to the Report prepared by the United Nations, Department of Economic and Social Affairs ”World Population Prospects 2019” also in African countries (Sub-Saharan Africa), the percentage of the population over the age of 65 will increase by more than 200% by 2050 compared to 2019. Similar data were obtained for Asia, Latin America and Australia. Therefore, it is important to create modern wound dressings that will be used in an emergency, reconstructive medicine, etc., but also in terms of palliative care ensuring the improvement of the quality of life in an aging society. Research is currently being carried out to insert a sensor into the dressing to monitor the wound healing process while maintaining the most important requirements for dressings (Figure 7).

**Figure 8:**
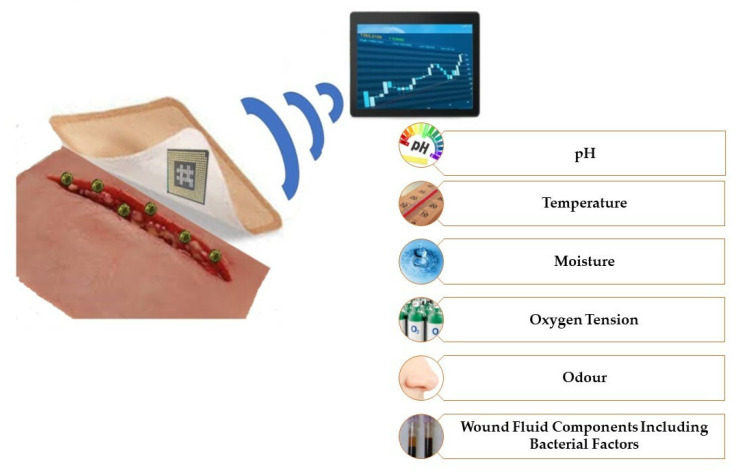
The possibility to control various wound parameters by sensors.

The dressing, apart from the expected properties presented above, should demonstrate more advanced capabilities. Society and medicine expect new technologies that will be a response to current problems such as diabetic foot, pressure ulcers, ulcerated wounds, burns, and varicose wounds. One such possibility is the use of sensors that control various parameters. Using sensors in dressings, it is possible to control various physical or chemical parameters of the wound, i.e., pH, temperature, moisture, and exudates (Figure 8) [33].

The use of a sensor in dressings allows for control of the wound-healing process. The control may concern various important parameters of the wound, changes may indicate the healing stage, the presence of pathogens and necrotic processes. The sensor can continuously monitor the condition of the wound, which is especially important in the case of difficult wounds, such as ulcers, bedsores and diabetic foot. Monitoring reduces unnecessary and excessive changes in the dressing, which may affect, for example, fluctuations in moisture, irritation of tissues, and loss of barrier [144]. Table 3 shows examples of parameters that can be monitored by indicators with information about their physiological significance for the wound.

**Figure 9:**
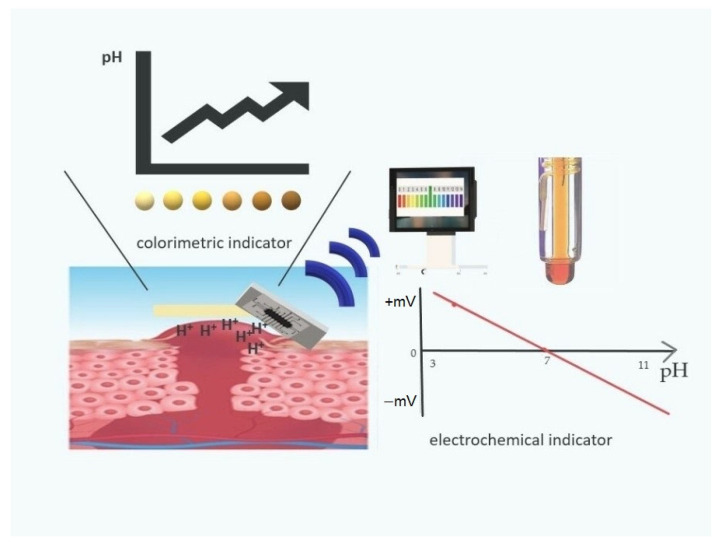
Colorimetric and electrochemical indicators.

The most frequently monitored physical parameter is pH. To monitor this parameter, scientists are developing indicators with different modes of operation, e.g., colorimetric and electrochemical indicators (Figure 9). 

Nischwitz et al. [149] produced composite nanofibrillar cellulose with the pH indicator GJM-534 (4-[4-(2-hydroxyethanesulfonyl)- phenylazo]-2,6-dimethoxyphenol) (BNC-SENS). Depressing allows visual observation of changes in the wound in the range of pH 7–10. This research aimed to create a sterile and easy-to-use method for monitoring pH without removing the dressing. Dressings were made of epiphyte hydro (alloplastic epidermal substitute), a translucent biomaterial made of BNC (biotechnologically generated nanocellulose with a water content of at least 95%) [149]. However, continuous electrochemical monitoring is also an important solution. Changes in pH lead to the absorption of hydrogen ions, causing valency changes in the oxygen atoms of the metal oxide [150]. Mostafalu, P et al. made a pH sensor consisting of a working electrode and a solid-state Ag/AgCl reference electrode [151]. Whereas Sharp, D. prepared the printed carbon electrodes and Melai et al. electrode with graphene oxide (GO) [152,153]. Moisture indicator dressings are also important. Milne, S.D. et al. proposed a wound sense moisture sensor applied directly to the wound. The moisturizing control limited the unnecessary, excessive changes in the dressing. The wound was intact and the healing process was more effective [154].

As pH stimulates volumetric changes, the amount of water present in the polymer matrix differs from 20% to 99% based on the pH that the hydrogel is immersed in. The main key to exhibiting pH sensitivity is the presence of a backbone polymer that contains weak acidic or basic groups, which become more ionized based on changes in pH. Depending on the pH of the solution the number of carboxylic ions changes, which influences the refractive index of the hydrogel [155]. 

#### 5.2.1. Sweat Measurement by Wearable Sensors 

Different variations in sweat pH can be correlated with different pathological and physiological conditions. Usually, sensitive elements are trapped in a polymeric network (hydrogel). The responsiveness can be triggered by external influence for example reversible swelling or shrinking, which is caused by changes in the equilibrium of electrostatic forces. This is also sensitive to changes in pH, which also alternates caused by different diseases. Such a responsive material can be prepared by co-polymerization of 10 kDa poly(ethylene glycol)-diacrylate (PEG-DA) macromer with 2-carboxyethyl acrylate (CEA), resulting in a soft copolymer and, more importantly, it is pH sensitive. pH sensitivity is due to the carboxylic group of CEA, which is protonated in acidic conditions and deprotonated in a basic environment. Resulting in repulsion or attraction of chains. 

#### 5.2.2. Epidermal Wounds 

Healthy skin exhibits slightly acidic properties and the pH of the wound varies from alkaline to neutral around 7.15 to 8.93. Tamayol conducted experiments on alginate-based microfibers that are loaded with pH-responsive silica beads that change color, depending on the pH dye. The presence of hydrogel in healing wounds is beneficial as it maintains a moist environment. Changes in dressing color allow for monitoring of the healing process. 

Next to monitoring, it was determined that it is possible to enhance wound healing. Hydrogel made of poly(N-isopropyl acrylamide-co-acrylic acid) via radical copolymerization which was loaded with bovine serum albumin (BSA), which fills the role of growth factor, was constantly released at increased pH. Such a hydrogel may as well be loaded with antibiotic agents, and combined with the monitoring of bacterial infections with dye, results in a very effective way to enhance wound treatment. 

Kwon et al. fabricated hydroxyethyl cellulose hydrogel with hyaluronic acid for treating skin lesions. Based on swelling and shrinking properties thanks to changes in pH, the primarily captured drug was slowly released. The efficiency of such drug release was estimated to be more than 70% at pH 7.

#### 5.2.3. Drug Delivery 

pH-sensitive hydrogels have also been investigated in peptide drug delivery, which cannot be delivered in acidic regions. Consequently, hydrogel protection is a chance of successful delivery, without losing the drug. For this purpose, Yadav and Shivakumar used soluble water chitosan to deliver the drug to the intestines. It was found that the sensitivity of the hydrogel was strongly connected to the concentration of the crosslinking agent, which was carboxymethyl chitosan and Carbopol. This method was used in the prolonged releasing theophylline in the intestine for treating nocturnal asthma. A similar treatment was developed by Dai et al. for delivering the anticancer drug as the pH close to the tumor decreases below the physiological range. It was also proved that NIR irradiation can accelerate drug release. Considered for this role were two polymers: N-(2-hydroxypropyl) methacrylamide (HPMA) and DF-PEG-PAHy/BPNSs. 

Polyethylene glycol dimethacrylate (PEGDMA) and methacrylic acid (MAA) in a molar feed ratio of 1:2 with ammonium sulphate result in pH-sensitive hydrogel and near-infrared (NIR) light and showed ideal mechanical and swelling properties. The material was tested in vivo as drug delivery for chemotherapy treatment (doxorubicin (DOX)) the drug was efficiently contained in the hydrogel structure for cancer therapy and then released by changes in pH or NIR. 

Composite consisting of graphene oxide and para-aminosalicylic acid after lyophilization results in the air-dried hydrogel. This combination of polymers exhibited both pH-sensitive properties and different drug release profiles in neutral and acidic conditions. It is sensitive to changes in the blood pH as well as the internal pH of infected macrophages [155]. 

## 6. Perspectives

**Figure 10:**
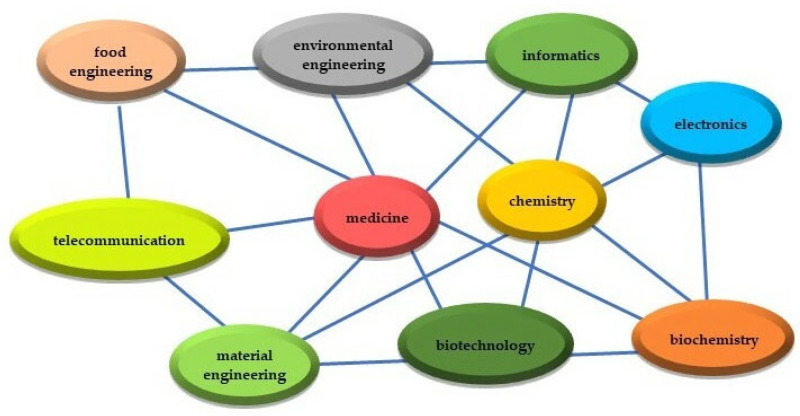
The most important scientific disciplines in biosensor applications.

The future of biopolymer composites with indicators that respond to a change in the biological environment seems to be still a long way off. Scientific disciplines permeate and complement what creates a space of great potential (Figure 10). The network of disciplines enables a response to current and future environmental and social problems, such as ocean and freshwater pollution, civilization diseases, an aging society and genetic diseases. The above problems are a stimulus for scientists, industry, non-governmental organizations, and maybe even governmental ones, to make indicator biocomposites not only in the space of science but to be commercialized.

**Figure 11:**
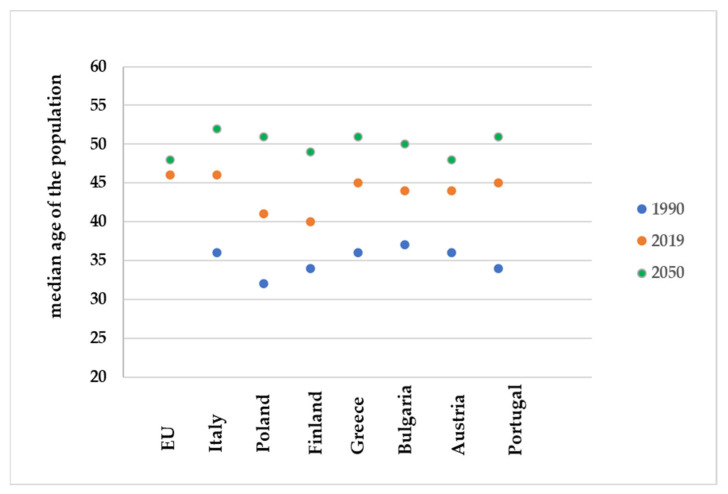
Median age of the population in the EU (Eurostat).

One of the driving challenges in developing new indicator composites is an aging society. All countries face major challenges to ensure that their health and social systems will be ready for demographic shifts. Every country in the world is experiencing growth in both the size and the proportion of older persons in the population. WHO warns of the global demographic trend, the pace of population aging is much faster than in the past. Between 2015 and 2050, the proportion of the world’s population over 60 years will nearly double from 12% to 22%. Statistical data clearly shows that in the EU we currently have a trend of an aging society (Figure 11).

**Figure 12:**
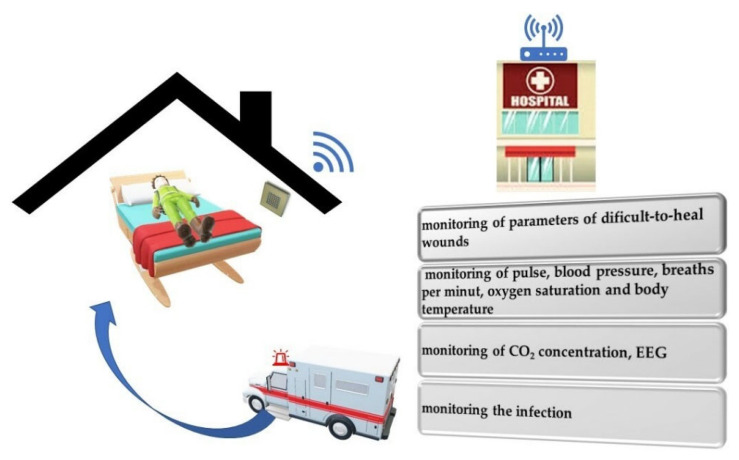
Control of the patient’s parameters at home.

The aging society will require the introduction of new medical technologies in hospitals and domestic use. Biosensors will transmit data from the patient’s home to the medical center, where they will be verified. Home care will be supervised, and the sensor system will send this information to the medical center in the event of any changes. The patient will be under constant supervision in a more favorable home environment. The future of biosensors will allow for extensive control of parameters at home and quick reaction of the control center to unfavorable changes Figure 12.

**Figure 13:**
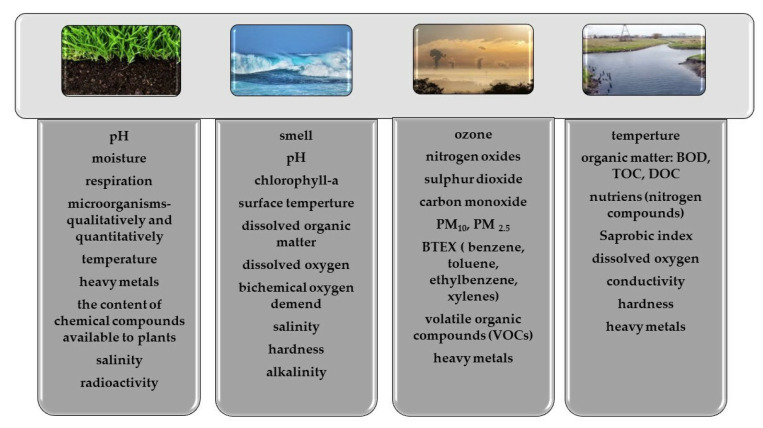
The exemplary parameters are to be monitored in various environments.

The control of the environment by biosensors will be highly desirable in the future. In 2022, an ecological disaster took place on the Odra River. Currently, golden algae are indicated as the indirect cause of the catastrophe. The immediate cause was the increase in salinity. However, the problem is more complex and is the result of several stress factors, all of which were caused by human activity. The disaster caused mass mortality in fish, mussels and water snails in August 2022. Now the biomass of the perished animals is being decomposed by bacteria, which is still dangerous for the river [156]. Biosensor systems that could continuously monitor the environment will be an important task in the coming years. Depending on the environment, monitoring may involve various parameters which are presented in Figure 13 [11,157,158,159,160,161,162].

## 7. Summary

Biopolymers are of great importance in the design of biocomposites with sensors for various applications, for example, to be used in medicine or the environment. The variety of properties of biopolymer materials allows for wide application possibilities in many branches of industry for example biosensors used in medicine. Biosensors are fast, portable systems that enable monitoring of the environment in which they are located. These materials play an important role in medical diagnostics and patient monitoring. Biosensors are suited to various diagnostic and real-time detection challenges owing to the use of biological molecules, tissues, and organisms. Medical biosensors are suitable for monitoring health parameters. These medical materials help biomedical engineers, researchers, molecular biologists, oncologists and clinicians with the development of point-of-care devices for disease diagnostics and prognostics. It also provides information on developing user-friendly, sensitive, stable, accurate, low cost and minimally invasive modalities which can be adopted from the lab to clinics.

Civilization diseases, i.e., diabetes, strokes, and diseases of the cardiovascular system, along with an aging society, require the use of new medical therapies. A future direction is composites based on biopolymers with sensors informing about physiological and chemical aspects. Such solutions will enable faster and more effective treatment of difficult-to-heal wounds, such as pressure ulcers, diabetic foot ulcers and wounds after extensive surgeries. They are necessary to improve patients’ functioning and survival. Scientists have conducted numerous studies and the development of medicine has long exceeded the wildest expectations of science fiction authors. This level of science allows us to design very advanced sensory technologies. The only barrier is the economic aspect, such materials are expensive and difficult to access. Nevertheless, their development is certain, and the costs will be reduced in the future. It is difficult to calculate human health and life, and therefore medical care should use new technologies.

## Figures and Tables

**Table 3:** Wound parameters and their physiological importance.

Parameter	Physiological Significance	Literature
**pH**	The pH value depends on the stage of the wound-healing process. A lower pH is optimal for wound healing and promotes collagen formation, increases the activity of fibroblasts and inhibits the proliferation of bacteria. Alkaline pH values (7–9) are typical of difficult-to-heal wounds, i.e., bedsores and diabetic foot.	[24,25]
**temperature**	Symptoms of a wound infection are elevated temperature. Temperature increases because of infection and inflammation.	[145,146]
**moisture**	The appropriate level of moisture accelerates healing and reduces the risk of tissue necrosis, helps maintain enzymatic balance and reduces pain. A too-dry wound environment can slow down the healing process and promote scarring. A dry wound is often covered with fibrin deposits, the fundus granulation fibrosis, and the wound edges do not skin properly. Too much moisture in the environment exposes the skin to damage, promotes the spread of infections and causes maceration of the wound edges.	[27,28]
**oxygen tension**	Oxygen supply is critical for all aspects of wound healing and the biochemical energy supply. The loss of parts of the vascular network and some necrotic tissue affects the oxygen tension. Hypoxia as a feature of chronic wounds.	[29,30,31]
**odor**	The sources of an unpleasant odor there are necrotic tissues of the skin, subcutaneous tissue, muscles, sometimes bones and wounds infected with anaerobic and aerobic bacteria. The products of tissue decomposition that are directly responsible for the odors are volatile short-chain fatty acids.	[147,148]
**wound fluid components, including bacterial factors**	Depending on the condition of the wound, various substances can be secreted from the wound by the body, e.g., markers of inflammation: cytokines, neuropeptides, hydroxyl radical, hydrogen peroxide. Various substances can be secreted by microbes, e.g., uricase, alpha-Hemolysin, Rhamnolipid B, methyl ketones, particularly 2-nonanone, and 2-undecanoate.	[32,33]

## Data Availability

Not applicable.
